# Use of *Phalaris canariensis* Extract as CO_2_ Corrosion Inhibitor of Brass

**DOI:** 10.3390/ma18153449

**Published:** 2025-07-23

**Authors:** Edgar Salazar-Salazar, Dante Guillermo Gutierrez-Granda, Earvin Galvan, Ana Karen Larios-Galvez, America Maria Ramirez-Arteaga, Roy Lopez-Sesenes, Alfredo Brito-Franco, Jesus Porcayo-Calderon, Jose Gonzalo Gonzalez-Rodriguez

**Affiliations:** 1 Facultad de Ingeniería, Universidad Nacional Autonoma de Mexico, Ciudad Universitaria, Ciudad de Mexico 04510, Mexico; salelas@gmail.com; 2 Centro de Investigacion en Ingenieria y Ciencias Aplicadas, Universidad Autonoma del Estado de Morelos, Av. Universidad 1001, Cuernavaca 62209, Mexico; dante.gutierrez.ism@gmail.com (D.G.G.-G.); earvin.galvanb@uaem.edu.mx (E.G.); karengalvex@gmail.com (A.K.L.-G.); alfredo.britof@uaem.edu.mx (A.B.-F.); 3 Facultad de Ciencias Quimicas e Ingenieria, Universidad Autonoma del Estado de Morelos, Av. Universidad 1001, Cuernavaca 62210, Mexico; america.rmz@uaem.mx (A.M.R.-A.); rlopez@uaem.mx (R.L.-S.); 4 Departamento de Ingeniería Química y Metalurgia, Universidad de Sonora, Hermosillo 83000, Mexico; jporcayoc@gmail.com

**Keywords:** brass, acidic corrosion, green inhibitor, *Phalaris canariensis*

## Abstract

In this study, the corrosion inhibition of a *Phalaris canariensis* extract on brass in a CO_2_-saturated 3.5% NaCl solution is evaluated with the aid of potentiodynamic polarization curves and electrochemical impedance spectroscopy tests. The results indicate that the *Phalaris canariensis* extract is an excellent CO_2_ corrosion inhibitor with an efficiency that increases with its concentration, reaching its maximum value of 99% with an inhibitor concentration of 100 ppm, decreasing the corrosion current density by more than two orders of magnitude. The addition of the *Phalaris canariensis* extract increased the pitting potential, decreased the passive current density values, and affected cathodic reactions, behaving as a mixed type of inhibitor. The corrosion process was under charge transfer control, and it was neither affected by the addition of the inhibitor nor by the elapsing time. The main compounds found in the *Phalaris canariensis* extract included antioxidants such as palmitic and oleic acids.

## 1. Introduction

Copper and its alloys, such as brass, possess excellent electrical and thermal properties as well as good mechanical properties and corrosion resistance, making them candidates for many applications in industry, such as drinking water distribution systems, power stations, seawater desalination, submarine and ship construction, pipelines, oil–water separation processes, electronics, etc. Despite the high corrosion resistance of copper and brass, they are susceptible to different types of corrosion when exposed to some environments, especially the selective corrosion of zinc in brass [1,2,3,4]. It has been proven that one of the most efficient ways to minimize copper or brass corrosion is by using corrosion inhibitors, especially heterocyclic compounds that contain chemical elements such as P, S, N, and O in groups such as amines, azoles, Schiff bases, imidazolines, amides, and their derivatives, which can form a layer of protective corrosion products on the metal surface [5,6]. However, these organic compounds are expensive, toxic, and harmful to human people and the environment, and thus, a lot of research has been conducted on the use of more environmentally friendly corrosion inhibitors [7,8,9,10,11,12,13,14,15,16,17,18,19,20]. Thus, some green inhibitors such as palm oil [7,12], *Aegle marmelos* pulp [8], shrimp shell waste [9], carbon dots [11], expired ciprofloxacin [13], *Thymus vulgaris* [15], coffee waste [16], and garlic [18] have been evaluated for copper and brass in environments such as acid rain [7,12,13,14], H_2_SO_4_ [8], NaCl [9,10,16,19], HCl [14], and H_2_NO_3_ solutions [17,18]. However, there is very little information about the CO_2_ corrosion of copper or brass [21,22,23].

In power stations, brass is used in various components like valves, fittings, connectors, and steam condensers due to its resistance to corrosion, especially in water or steam systems. Some pump components, such as impellers and housings, may be made of brass to resist wear and tear as well as corrosion from fluids like water or oil. In thermal power plants, particularly coal-, gas-, or oil-fired stations, CO_2_ is a byproduct of the combustion of fossil fuels. This CO_2_ is present in the flue gases that are emitted during the burning of fuels [24]. While the CO_2_ itself is usually not directly mixed with the station working fluids, it may dissolve in water or other fluids used in cooling systems, leading to an increase in CO_2_ concentration in these fluids [25]. If the plant uses a wet cooling tower, the flue gases may carry CO_2_ into the cooling water, either through direct contact with the air or through leakage from systems like the condenser or scrubbers. When CO_2_ dissolves in water, it forms carbonic acid (H_2_CO_3_), which can lower the pH of the cooling or condensate water [26]. This can lead to corrosion in pipes, heat exchangers, and other parts of the cooling system if not properly controlled. In power stations that use steam turbines, the water in the steam cycle may absorb CO_2_ from the combustion gases, especially if the boiler is not fully sealed or if there is any leakage. Over time, CO_2_ can build up in the steam and condense back into water within the system [27].

*Phalaris canariensis* is a species of grass native to the Mediterranean region, including the Canary Islands (hence the name), which grows up to 1 m (3 feet) tall; it has erect, hairless stems and narrow, flat leaves, with small, 4–5 mm long, glossy, and brownish seeds, commonly used as birdseed. Although it is used mainly as a bird food, it has gained importance for human consumption due to benefits such as antioxidant, antihypertensive, and antidiabetic properties due to the presence of amino acids, proteins, minerals, and a variety of antioxidants such as flavonoids, vitamin E, and fatty acids such as oleic, palmitic, and linoleic acids [28,29,30]. Thus, the aim of this work is to evaluate the environmentally friendly corrosion inhibitor, *Phalaris canariensis*, for the CO_2_ corrosion of brass.

## 2. Experimental Procedure

### 2.1. Testing Material

The material used in the present research work was commercial brass containing 68.3 (wt. %) Cu-31.0 Zn-0.3 Fe-0.1 Al-0.3 Pb in the form of bars measuring 6.0 mm in diameter; 10.0 mm long samples were cut and embedded in commercial resin, polished with 600 grade emery paper, washed with acetone, and blown with warm air.

### 2.2. Synthesis of the Inhibitor

For the extraction of the inhibitor, the Soxhlet method was used using hexane as the solvent. Although different solvents were considered, such as hexane, chloroform, and methanol, according to Maharaj et al. [31], who worked with leaves of Mangifera indica, the hexane extract had significant antioxidant activity, while methanolic extracts exhibited antibacterial activity. For this reason, hexane was used in this work. *Phalaris canariensis* seeds were crushed, and 400 g of this powder was placed together with 100 mL of hexane in flasks at 68.7 °C for 24 h, as recommended by Maharaj et al. [31]. The remaining solvent was evaporated in a rotary evaporator, leaving only the extracted *Phalaris canariensis* oil. Chemical characterization of the extract was performed by gas chromatography–mass spectrometry (GC-MS) analysis by using an Agilent Technologies machine (Santa Clara, CA 95051, USA) equipped with an HP-5ms capillary column (30 m, 0.25 mm i.d., 0.25 µm film thickness) coupled to a 5973N mass spectrometer as a detector.

### 2.3. Working Electrolyte

A solution made of 3.5 (wt.%) and bubbled with CO_2_ was the electrolyte. For this, a 3.5% NaCl solution was prepared by using analytical-grade reagents. This naturally aerated solution was bubbled for two hours with CO_2_ gas and kept bubbling during the whole test. The initial oxygen concentration was not measured, and no nitrogen gas was bubbled into the solution to eliminate the oxygen present, but it was expected that CO_2_ would displace other gases, including O_2_, from the liquid. This reduces the partial pressure of oxygen and encourages its degassing. The conductivity and pH values of this de-aerated solution were 52 mS/cm and 6.1, respectively. A three-electrode glass electrochemical cell was used for this purpose, using a Silver/Silver Chloride reference electrode and a 6.0 mm graphite counter electrode. An inhibitor was added to this solution by appropriate dilutions.

### 2.4. Electrochemical Techniques

Potentiodynamic polarization curves (PPCs) and electrochemical impedance spectroscopy tests (EIS) were used to evaluate the inhibitor. Prior to these tests, the open-circuit potential value, OCP, was monitored over 1800 s. The PPC tests were started in the cathodic branch, applying a potential of 700 mV more cathodic than the free corrosion potential value, E_corr_, moving into the anodic direction at a scan rate of 0.3 mV/s, and stopping the test 700 mV more anodic than E_corr_. To calculate the corrosion current density value, I_corr_, the Tafel extrapolation method was used, but when a passive zone was found, we extrapolated the passive current density value. This is a standard procedure normally performed in the literature [3,5,6,8,9,10,11,12]. The inhibitor efficiency value, I.E., was calculated as follows:

I.E. (%) = (Ι_corr1_ − Ι_corr2_)/Ι_corr1_ × 100 (1)

where I_corr2_ and I_corr1_ are the corrosion current density values with and without the inhibitor, respectively, and the metal fraction covered by the inhibitor, θ, was calculated by using

θ = (Ι_corr1_ − Ι_corr2_)/Ι_corr1_ (2)

The EIS measurements were carried out with the E_corr_ value by applying an AC voltage signal of ±10 mV in a frequency range of 100 kHz to 0.01 Hz over 24 h. The morphology of the corroded specimens was analyzed using a JEOL JSM-IT500 Scanning Electronic Microscope, SEM,(JEOL Mexico, Mexico City) which was coupled to an Oxford energy dispersive spectrometer, EDS, to carry out a semi-quantitative chemical analysis of the corrosion products on the top of the specimens.

## 3. Results and Discussion

### 3.1. Metal Characterization

**Figure 1:**
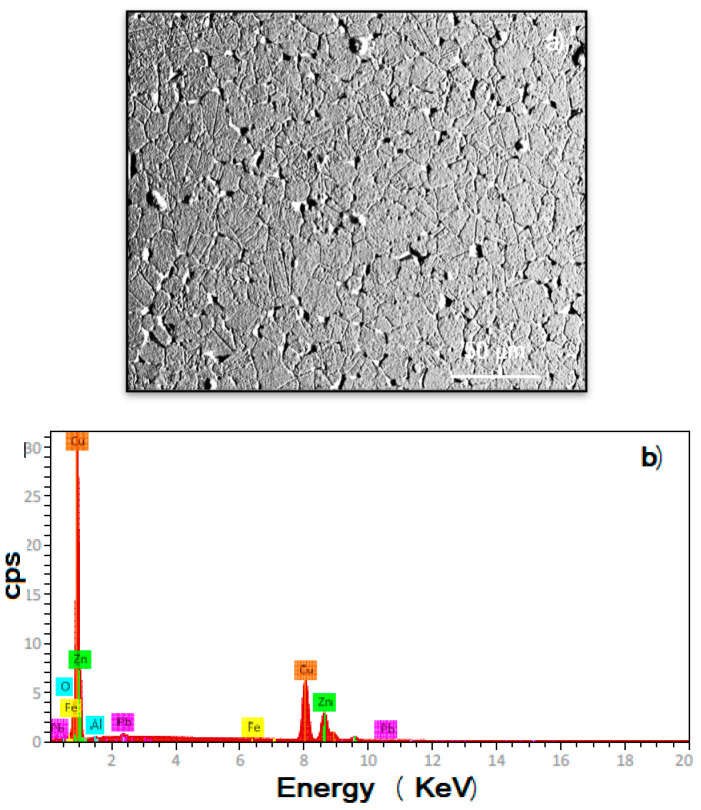
(**a**) SEM micrograph of tested brass showing its microstructure and (**b**) EDS chemical elemental analysis.

The microstructure and chemical analysis of tested brass are shown in Figure 1. The microstructure consists of polygonal, equiaxed grains, with a heterogeneous grain size ranging between 10 and 30 μm, with the existence of some subgrains, as shown in Figure 1a. Some precipitates can be observed along the grain boundaries, which contained Pb, Fe, and Al. EDX corrobo rated that the major chemical elements are Cu (around 65%) and Zn (around 34%), with minor traces of Fe, Pb, and Al. It is very important to note that these particles are at sites where localized types of corrosion such as pitting can take place.

### 3.2. Chemical Analysis of the Inhibitor

As reported elsewhere [32], Table 1 gives the chemical composition of the *Phalaris canariensis* extract consisting of fatty acids, with the most abundant being n-hexadecanoic (palmitic), oleic, and 17-octadecynoic (stearolic) acids. It is not possible to know which one is active from the point of view of corrosion inhibition, since each one of them has some antioxidant properties, but the three most abundant have been reported as good corrosion inhibitors. Palmitic acid has no double bonds, whereas oleic acid has double bonds in its carbon chain, giving antioxidant properties to these compounds, whereas the former gives more stability to its molecular structure. Double bonds are more chemically reactive than single bonds. It was also reported the presence of a C-H bond, as well as a C=O carbonyl group and C-O and C=C bonds. The combination of these functional groups as well as the double bonds mean that these fatty acids can act as corrosion inhibitors. In fact, both oleic and palmitic acids have been studied as corrosion inhibitors for carbon steel in CO_2_ environments [33,34] and in acidic media [35,36,37]. More inhibitor concentrations are needed to calculate an adsorption isotherm, but in [32] it was shown that for 314-type stainless steel, this extract adsorbs following a Temkin type of isotherm.

### 3.3. Open Circuit Potential

**Figure 2:**
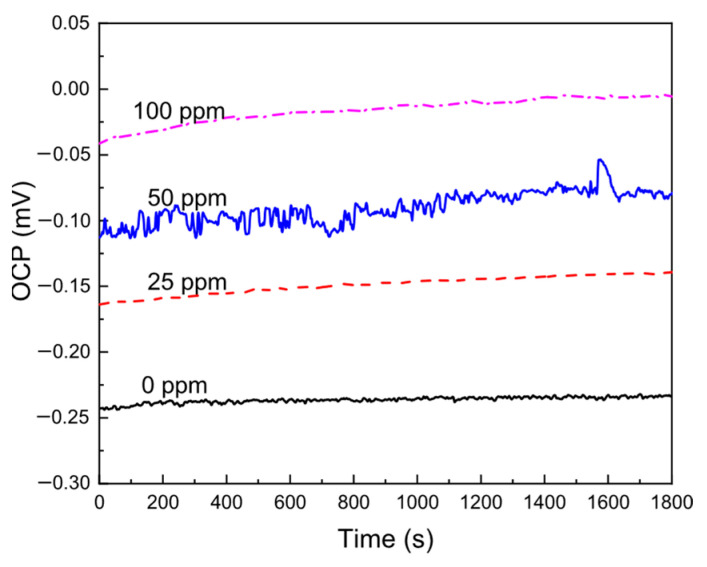
Variation in the OCP value for brass in a CO_2_-saturated 3.5% NaCl solution at different concentrations of *Phalaris canariensis* extract.

The values of the OCP at different concentrations of the *Phalaris canariensis* extract for brass in the CO_2_-saturated 3.5% NaCl solution are depicted in Figure 2, where it can be seen that as the *Phalaris canariensis* extract increases, the OCP value is made nobler. We can see that the most active OCP value was found without addition of the inhibitor, and the most positive OCP value was obtained with the addition of 100 ppm of *Phalaris canariensis* extract. This shift in the OCP value is due to the adsorption of the *Phalaris canariensis* extract to form a protective layer of corrosion products. The main corrosion products reported for brass CO_2_ include CuO, Cu_2_O, CuCO_3_ and Cu(OH)_2_ [21,38], which are very protective, and this is the reason why the OCP value in these conditions is quite stable. As soon as the inhibitor was added to the solution, this was adsorbed onto the metal surface to form a protective layer, which, in combination with the compounds formed in the absence of the inhibitor, resulted in better protection given to the metal; the formed film that covered the alloy produces a shifting of the OCP value into the noble direction [22,23].

### 3.4. Potentiodynamic Polarization Curves

**Figure 3:**
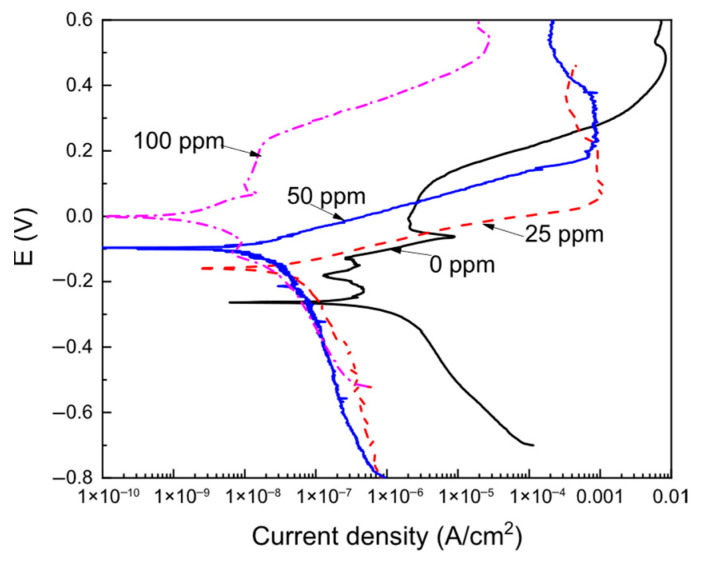
Potentiodynamic polarization curves for brass in a CO_2_-saturated 3.5% NaCl solution at different concentrations of *Phalaris canariensis* extract.

The potentiodynamic polarization curves for brass in the CO_2_-saturated 3.5% NaCl solution using different concentrations of the *Phalaris canariensis* extract are depicted in Figure 3. Extracted parameters such as E_corr_, I_corr_, anodic and cathodic Tafel slopes, inhibitor efficiency, and metal surface area covered by the inhibitor, θ, are given in Table 2. Figure 3 shows that the data in the uninhibited solution display an active–passive behavior; with the addition of either 25 or 50 ppm of inhibitor, the passive zone disappears, and it appears again when 100 ppm of inhibitor is added to the solution.

Anodic reactions involve the oxidation of Zn as Zn^2+^ as follows:

Zn → Zn^2+^+ 2e (3)

On the other hand, the cathodic reaction involves the transformation of dissolved CO_2_ into carbonic acid (H_2_CO_3_) according to [39]

CO_2_ + H_2_O → H_2_CO_3_ (4)

which leads to the formation of HCO_3_^−^, CO_3_^2−^ and H^+^ [40,41] according to

H_2_CO_3_ → H^+^ + HCO_3_^−^ (5)

HCO_3_^−^ → H^+^ + CO_3_^2−^ (6)

H^+^ + e^−^ → ½H_2_ (7)

In the absence of the inhibitor, two very narrow passive regions are observed at −0.240 and −0.185 mV, whereas a very wide zone that starts at −0.90 mV can be observed. As it was established above, the main corrosion products in the blank, uninhibited solution are CuO and Cu_2_O oxides, CuCO_3_ and Cu(OH)_2_ [21,38]. When the inhibitor is added, the passive zone formed by the adsorbed inhibitor onto the metal surface can be still observed, the E_corr_ value is shifted into the noble zone, and both the passive and corrosion current density values are decreased, as shown in Table 1. The pitting potential value, E_pit_, also moved towards nobler values, and the passive current density value decreased as the inhibitor concentration increased. Thus, we can say that the addition of the *Phalaris canariensis* extract improved the passive film properties. It can be seen that the I_corr_ value decreased as the inhibitor concentration increased, obtaining the lowest value with the addition of 100 ppm, which was nearly two orders of magnitude lower than that obtained for the uninhibited solution. Table 1 also shows that the inhibitor efficiency increases with the inhibitor concentration, reaching the highest value of 99% with 100 ppm of the *Phalaris canariensis* extract. Similarly, the metal fraction covered by the inhibitor, θ, increases with the inhibitor concentration, indicating that the corrosion inhibition is due to the adsorption of the *Phalaris canariensis* extract onto the metal surface. On the other hand, since there was a passive zone with properties improved by the addition of the inhibitor, no anodic Tafel behavior can be observed; however, the cathodic Tafel slope was affected by the addition of the inhibitor, affecting the cathodic reactions described above. Therefore, it can be said that *Phalaris canariensis* extract behaves as a mixed type of inhibitor.

### 3.5. Electrochemical Impedance Spectroscopy Tests (EIS)

**Figure 4:**
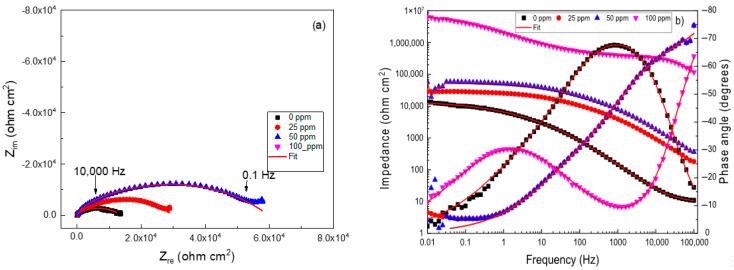
(**a**) Nyquist diagrams for brass immersed in a CO_2_-saturated 3.5% NaCl solution at different *Phalaris canariensis* extract concentrations: insert is the plot at 100 ppm, and (**b**) Bode plots for brass in for brass immersed in a CO_2_-saturated 3.5% NaCl solution at different *Phalaris canariensis* extract.

The EIS data in Nyquist and Bode formats for brass in the CO_2_-saturated 3.5% NaCl solution with different concentrations of the *Phalaris canariensis* extract are given in Figure 4. The Nyquist diagrams, shown in Figure 4a, display two capacitive loops [3,4,5], which means that the corrosion process is charge-transfer controlled [6,7]. As the concentration of the inhibitor increased, the same types of loops were displayed, which means that the addition of the inhibitor did not affect the corrosion mechanism. The diameter of the semicircle observed at high frequency values increases with the inhibitor concentration, reaching its highest value at an inhibitor concentration of 100 ppm. The Bode plots in Figure 4b show that the impedance increases with the inhibitor concentration, increasing nearly three orders of magnitude with the addition of 100 ppm of inhibitor; two different slopes can be observed in these plots, and thus, there is the existence of two time constants. The phase angle also increases with the inhibitor concentration, reaching its highest value at an inhibitor concentration of 100 ppm.

**Figure 5:**
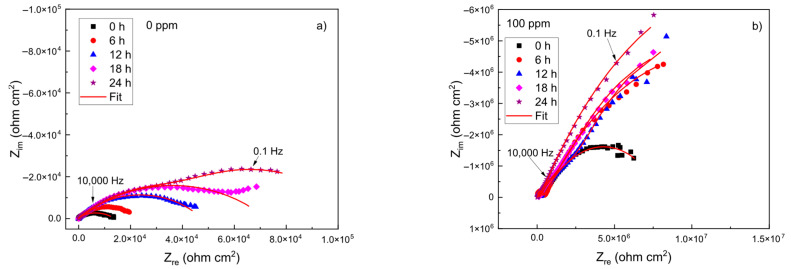
Nyquist diagrams at different exposure times for brass in a CO_2_-saturated 3.5% NaCl solution containing (**a**) 0 and (**b**) 100 ppm of *Phalaris canariensis* extract.

**Figure 6:**
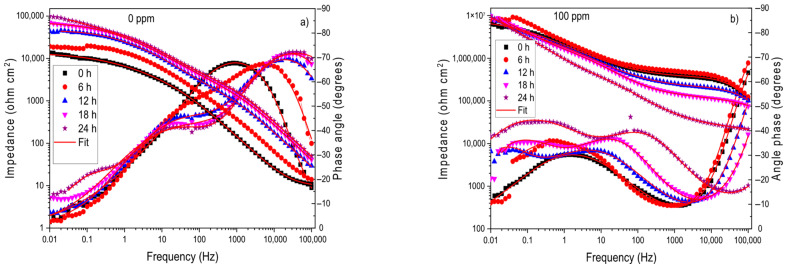
Bode diagrams for brass at different exposure times in a CO_2_-saturated 3.5% NaCl solution containing (**a**) 0 and (**b**) 100 ppm of *Phalaris canariensis* extract.

The evolution with time of the Nyquist and Bode plots for the uninhibited solution and the solution containing 100 ppm of inhibitor is shown in Figure 5 and Figure 6, respectively. For the uninhibited solution, the shape of the Nyquist plots changed as time elapsed during the first 18 h of testing, as shown in Figure 5a, since for times shorter than 18 h, a single capacitive semicircle was observed; however, for times longer than 18 h, two loops are observed: one capacitive, depressed loop at high- and intermediate-frequency values, related to the processes occurring across the double electrochemical layer, followed by a second one at lower frequencies, related to the formation of a protective layer such as copper carbonates/oxides. This behavior was observed in all the replicates. The phase angle showed a single peak for times shorter than 6 h; however, after 6 h of testing, two peaks appeared, as shown in Figure 6b, whereas the total impedance increased as time elapsed, with the existence of two slopes at all testing times and thus two different time constants.

For the solution containing 100 ppm of inhibitor, the Nyquist diagrams were not altered as time elapsed, which means that the corrosion mechanism remained unchanged, as shown in Figure 5b.

The total impedance increased in a slow fashion as time elapsed, and three different slopes were observed for all testing times; on the other hand, the phase angle value increased with time, with the existence of three peaks for all testing times, indicating the formation of a very protective corrosion product layer.

**Figure 7:**
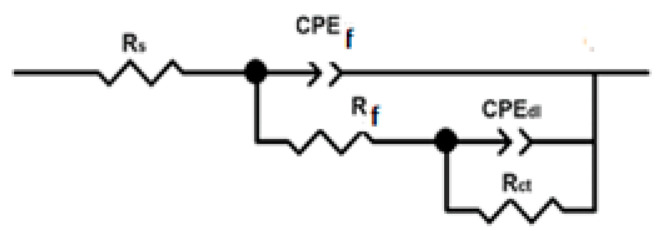
Electric circuits used to simulate the EIS data for brass in a CO_2_-saturated 3.5% NaCl+CO_2_ solution.

To simulate the EIS data, an electric circuit such as that shown in Figure 7 was used. In this figure, the solution, charge transfer, and oxide film resistances are represented by R_s_, R_ct_, and R_f;_ respectively; the double electrochemical layer and oxide capacitances are represented by C_dl_ and C_f_ As observed above, the observed loops in Figure 3, Figure 4 and Figure 5 are depressed due to surface heterogeneities or surface corrosion, so ideal capacitances are replaced by a constant phase element, CPE, which has an impedance, Z_CPE_, given by

(8) $$Z_{CPE}=Y_{0}^{-1}{\left(jw\right)}^{-n}$$

In this expression, a proportionality constant is given by *Y_0_*, the imaginary unit is *j*, the angular frequency is represented by *ω*, and the heterogeneity and roughness of the surface is denoted by n [42]. The presence of two CPE’s in Figure 7 is justified because the data displayed in Figure 3, Figure 4 and Figure 5 showed the existence of two different phase constants. In addition to this, another way to calculate the capacitance of the double electrochemical layer is by using the following expression [43,44]:

C_dl_ = (εε_0_/λ)A (9)

where ε_0_ and ε are the free space permittivity and the dielectric constant of that space, respectively, the thickness of the double layer is λ, and the electrode surface area is denoted by A.

The electrochemical parameters obtained by using the electric circuit shown in Figure 6 are summarized in Table 3, Table 4 and Table 5. Table 3 shows that as the *Phalaris canariensis* extract increases, the R_ct_ values and inhibitor efficiency increase, whereas the CPE_dl_ value decreases. In this case, inhibitor efficiency was calculated as follows:

(10) $$I.E.\left(\%\right)=\frac{R_{ct1}-R_{ct2}}{R_{ct1}}\times 100\;$$

where the charge transfer resistance in the presence and absence of the *Phalaris canariensis* extract are represented by R_ct1_ and R_ct2,_ respectively. As mentioned earlier, the adsorption of the inhibitor onto the brass surface and the formation of a protective corrosion product layer increases the inhibitor efficiency. The decreases in the corrosion rate, as given by the decrease in the I_corr_ value shown in Table 2, makes that the double layer conductivity decrease due to the reduction in the number of Cu^2+^ and Zn^2+^ ions crossing the double electrochemical layer, thus bringing an increase in its resistance represented by R_ct_. Also, when the inhibitor is added into the solution, the adsorbed water molecules on the alloy surface are displaced by those of the *Phalaris canariensis* extract; since the *Phalaris canariensis* extract molecules are much bigger than the water molecules, the double electrochemical layer thickness value, given by λ in Equation (9), increases, resulting in a decrease in the CPE_dl_ value. This is corroborated in Table 3, which shows that as the *Phalaris canariensis* extract concentration increases, the R_ct_ value increases, whereas the CPE_dl_ value decreases due to its adsorption onto the metal surface to form protective corrosion products and decrease the metal corrosion rate. Table 3 also shows that the film resistance values, R_f_, are bigger than those for R_ct_. An increase in the inhibitor concentration makes the value for R_f_ value increase, increasing the oxide film thickness. Parameter n can obtain values close to 1.0, indicating a low metal surface roughness because the metal resists the corrosive attack of the electrolyte; on the other hand, if parameter n has a value close to 0.5, the metal surface roughness is high because the metal is more susceptible to attack by the electrolyte. Table 3 shows that for the uninhibited solution, the n_dl_ value is 0.7, indicating a high metal surface roughness because the alloy is greatly corroded by the environment. This parameter increases as the inhibitor concentration increases, reaching a maximum value of 0.9 due to a low metal surface roughness because brass is much more resistant to corrosive environments.

On the other hand, for the uninhibited solution, shown in Table 3, the R_ct_ value did not change very much as the time elapsed, unlike the R_f_ values, which increased as the testing time elapsed by almost one order of magnitude, in agreement with the idea that under these conditions the oxides formed on top of the brass bring a decrease in its corrosion rate. However, for the test with the addition of 100 ppm, shown in Table 4, the R_ct_ value increased and the CPE_dl_ value decreased by almost one order of magnitude in both cases due to the inhibitor adsorption on the metal surface, with a decrease in the metal ions crossing the double electrochemical layer. Additionally, the film formed increased in thickness as the testing time elapsed, as evidenced by the increase in the R_f_ values.

### 3.6. Surface Characterization

**Figure 8:**
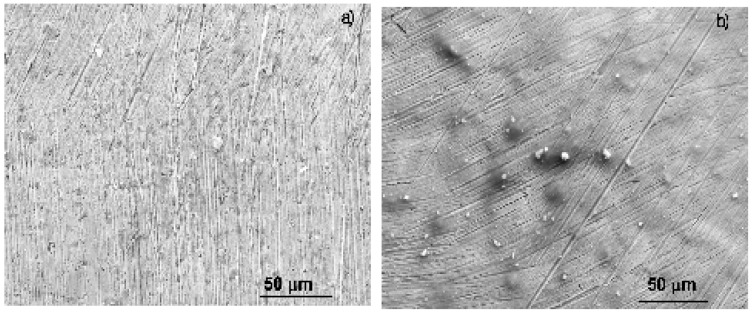
SEM micrographs of brass corroded in a CO_2_-saturated 3.5% NaCl+CO_2_ solution containing (**a**) 0 and (**b**) 100 ppm of *Phalaris canariensis* extract.

SEM micrographs of the corroded specimens in the uninhibited solution and in the presence of 100 ppm of the *Phalaris canariensis* extract are shown in Figure 8. For the specimen corroded in the uninhibited solution, shown in Figure 8a, the surface shows the presence of abrading marks together with numerous, small pits, indicative of the rupture of a passive layer formed on top of the alloy. The effect of the corrosive environment is clear, since a rough, corroded surface can be observed. On the other hand, regarding the specimen corroded in the presence of *Phalaris canariensis,* shown in Figure 8b, the abrading marks are still present, but a smoother surface without evidence of corrosion can be observed; however, some pits can be observed in a much lower amount than those present in the uninhibited solution, indicative that the passive layer formed on top of the alloy is more protective and less susceptible to disruption by aggressive ions present in the electrolyte.

**Figure 9:**
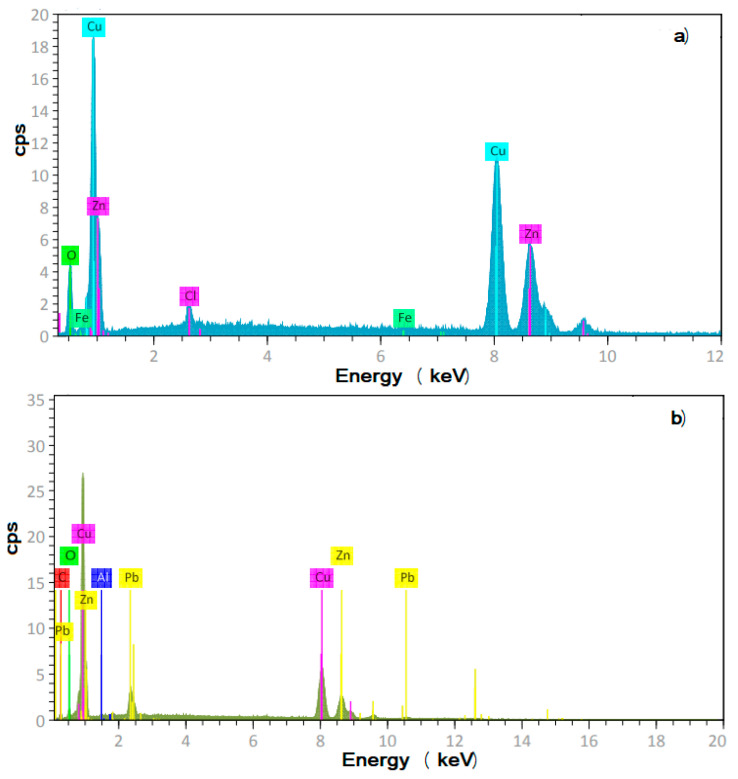
EDS chemical analysis of corroded brass in a CO_2_-saturated 3.5% NaCl+CO_2_ solution containing (**a**) 0 and (**b**) 100 ppm of *Phalaris canariensis* extract.

The EDS chemical microanalysis of the corroded samples in the absence and presence of the *Phalaris canariensis* extract is shown in Figure 9. The presence of chemical elements from the alloy and those from the corrosive environment such as Cl can be seen. However, the semi-quantitative results indicated that the Zn contents in both specimens were reduced from 34% down to 22 and 27% for specimens corroded in the absence and presence of the *Phalaris canariensis* extract, respectively, indicating dezincification of the alloy. On the other hand, for the specimen corroded in the presence of the inhibitor, the chemical analysis showed the presence of C, which comes from organic molecules present in the *Phalaris canariensis* extract. This is evidence that the inhibitor is adsorbed onto the metal surface to form a protective layer of corrosion products. This can be explained in terms of the *Phalaris canariensis* adsorption onto the metal surface replacing the water molecules adsorbed onto the metal surface according to the following expression:

Inh_sol_ + H_2_O_ads_ → Inh_ads_ + H_2_O_sol_ (11)

where Inh_sol_ and Inh_ads_ represent the inhibitor in the solution and adsorbed onto the steel surface, whereas H_2_O_sol_ and H_2_O_ads_ represent the water molecules in the solution and adsorbed onto the metal, respectively. Once the inhibitor is adsorbed on the metal surface, it forms a layer of protective corrosion products. When the passive film is beginning to form, some places on the alloy are not protected, leaving some active sites, or it is disrupted by the precipitates on the metal surface, shown in Figure 1, and a localized type of corrosion such as pits takes place, as observed on Figure 8.

## 4. Conclusions

The study of a *Phalaris canariensis* extract as a CO_2_-corrosion inhibitor for brass has been carried out. Polarization curves showed that even in the absence of the inhibitor, there is the presence of a passive layer on top of the alloy; the passive current density value decreased, whereas the pitting potential value increased with the addition of the *Phalaris canariensis* extract. For this, and since the cathodic Tafel layer was affected by the inhibitor, it can be said that the *Phalaris canariensis* extract behaves as a mixed type of inhibitor. The *Phalaris canariensis* extract was an excellent corrosion inhibitor since its adsorption on to the alloy surface formed a protective passive layer with an efficiency value that increases with increasing its concentration, obtaining a maximum efficiency of 99% with the addition of 100 ppm of inhibitor. The corrosion mechanism was under charge transfer control, and it was not affected by the addition of the inhibitor or by the elapsing time. The adsorption of the inhibitor onto the alloy surface to form a protective passive layer made the charge transfer resistance increase and the double layer capacitance decrease. This was due to the existence of antioxidant compounds such as palmitic and oleic acids on the extract.

## Figures and Tables

**Table 1:** GC-MS analysis of *Phalaris canariensis* extract [32].

RT (min)	Component	Chemical Formula	Area (%)
9.02	Oxirane, octyl	C_10_H_20_O	4.69
9.56	Biphenylene, 1,2,4a,4b,7,8,8a,8b-octahydro-	C_12_H_16_	3.53
0	Biphenylene, 1,2,4a,4b,7,8,8a,8b-octahydro-	C_12_H_16_	4
11.39	13-Tetradecenal	C_14_H_26_O	3.49
11.86	2,4-Decadienal, (E,E)-	C_10_H_16_O	6.17
12.2	2,4-Decadienal, (E,E)-	C_10_H_16_O	6.82
12.81	cis-1,2-Cyclododecanediol	C_12_H_24_O_2_	3.52
13.34	17-Octadecynoic acid	C_18_H_32_O_2_	3.78
19.9	n-Hexadecanoic acid	C_16_H_32_O_2_	60.2
21.66	Oleic Acid	C_18_H_34_O_2_	100

**Table 2:** Electrochemical parameters obtained from potentiodynamic polarization curves.

C_inh_ (ppm)	E_corr_ (V)	I_corr_ (A/cm^2^)	β_a_ (V/dec)	β_c_ (V/dec)	I.E. (%)	θ
0	−0.265	3.7 × 10^−7^	------	270	-----	---
25	−0.170	7.3 × 10^−8^	45	520	81	0.81
50	−0.100	3.0 × 10^−8^	55	540	91	0.91
100	−0.005	0.0 × 10^−9^	------	555	99	0.99

**Table 3:** Electrochemical parameters used to simulate the EIS data using different *Phalaris canariensis* extract concentrations.

C_inh_ (ppm)	R_ct_ (ohm cm^2^)	CPE_dl_ (F cm^−2^)	n_dl_	R_f_ (ohm cm^2^)	CPE_f_ (F cm^−2^)	n_f_	I.E.(%)
0	1777	$4.74\;\times \;10^{-6}$	0.7	10,503	$6.90{\;\times \;10}^{-5}$	0.7	----
25	6959	$2.52\;\times \;10^{-7}$	0.8	24,568	$4.37{\;\times \;10}^{-5}$	0.8	77
50	12,578	$1.17\;\times \;10^{-7}$	0.8	55,216	$2.29{\;\times \;10}^{-5}$	0.8	88
100	3.6 $\times {\;10}^{5}$	$6.5\;\times \;10^{-11}$	0.9	9$.8\times 10^{6}$	$1.90\;{\times \;10}^{-6}$	0.9	99

**Table 4:** Electrochemical parameters used to simulate the EIS data for the uninhibited solution.

Immersion Time (h)	R_ct_ (ohm cm^2^)	CPE_dl_ (F cm^−2^)	n_dl_	R_f_ (ohm cm^2^)	CPE_f_ (F cm^−2^)	n_f_
0	1777	$4.74\;\times \;10^{-6}$	0.7	10,503	$6.90\;{\times \;10}^{-5}$	0.7
6	1976	$1.3\;\times \;10^{-6}$	0.8	22,105	$1.3\;\times \;10^{-5}$	0.8
12	2125	$8.3\;\times \;10^{-7}$	0.8	46,186	$8.3\;\times \;10^{-6}$	0.8
18	2468	$6.2\;\times \;10^{-7}$	0.9	56,360	$6.2\;\times \;10^{-6}$	0.9
24	2797	$3.7\;\times \;10^{-7}$	0.9	90,613	$3.7\;\times \;10^{-6}$	0.9

**Table 5:** Electrochemical parameters used to simulate the EIS data for the solution containing 100 ppm of the inhibitor.

Immersion Time (h)	R_ct_ (ohm cm^2^)	CPE_dl_ (F cm^−2^)	n_dl_	R_f_ (ohm cm^2^)	CPE_f_ (F cm^−2^)	n_f_	I.E. (%)
0	3.6$\;\times \;10^{5}$	$6.5\;\times \;10^{-11}$	0.9	9.8$\;\times \;10^{6}$	$1.9\;{\times \;10}^{-6}$	0.9	99
6	$4.6\;\times \;10^{5}$	$1.6\;\times \;10^{-7}$	0.5	1.5$\;\times \;10^{7}$	$7.8\;\times \;10^{-7}$	0.9	99
12	$2.1\;\times \;10^{5}$	$1.7\;\times \;10^{-7}$	0.5	$2.0\;\times \;10^{7}$	$4.8\;\times \;10^{-7}$	0.9	99
18	$1.9\;\times \;10^{5}$	$1.1\;\times \;10^{-7}$	0.6	$2.8\;\times \;10^{7}$	$2.9\;\times \;10^{-7}$	0.9	99
24	$3.7\;\times \;10^{6}$	$1.4\;\times \;10^{-7}$	0.6	$5.1\;\times \;10^{7}$	$1.2\;\times \;10^{-7}$	0.9	99

## Data Availability

The original contributions presented in this study are included in the article. Further inquiries can be directed to the corresponding author.
